# A comparative analysis of movement and physical activity in early childhood teacher education policy in five Nordic countries

**DOI:** 10.3389/fspor.2024.1352520

**Published:** 2024-04-05

**Authors:** Lars Breum Christiansen, Jan-Eric Ekberg, Anne Soini, Robert Larsen, Gudrún Kristjánsdóttir, Karsten Froberg, Ann-Christin Sollerhed, Arja Sääkslahti, Ingunn Fjørtoft, Rúnar Vilhjálmsson, Line Grønholt Olesen

**Affiliations:** ^1^ Department of Sports Science and Clinical Biomechanics, Faculty of Health Sciences, University of Southern Denmark, Odense, Denmark; ^2^ Department of Sport Sciences, Malmö University, Malmö, Sweden; ^3^ Department of Education, Faculty of Education and Psychology, University of Jyväskylä, Jyväskylä, Finland; ^4^ Department of Sports, Physical Education and Outdoor Studies, Faculty of Humanities, Sports and Educational Science, University of South-Eastern Norway, Notodden, Norway; ^5^ Faculty of Nursing and Midwifery, School of Health Sciences, University of Iceland, Reykjavik, Iceland; ^6^ Faculty of Teacher Education, Kristianstad University, Kristianstad, Sweden; ^7^ Faculty of Sport and Health Sciences, University of Jyvaskyla, Jyväskylä, Finland; ^8^ Steno Diabetes Center Aarhus, Aarhus University Hospital, Aarhus, Denmark

**Keywords:** education policies, preschool, early childhood education, international comparison, physical development and movement

## Abstract

**Introduction:**

The aim of this study is to investigate the integration of movement and physical activity (MoPA) within Early Childhood Teacher Education (ECTE) policies across Denmark, Finland, Iceland, Norway, and Sweden. This knowledge can inform the development of ECTE policies and practices that promote MoPA in Early Childhood Education and Care (ECEC) in Nordic countries and other countries worldwide.

**Methods:**

In this study, a Nordic cross-national network of researchers collaborated in investigating policy documents at the national and university levels, which govern the education of ECEC teachers. This study was inspired by the Non-affirmative Theory of Education, which provides a framework for understanding the various influences on curricular development in higher education. Based on this, a four-step comparative analytical process of national and university documents across the Nordic countries was conducted. It included keyword search for MoPA related courses and a qualitative description of MoPA in ECTE. Thus, a combination of investigations of policy documents at the national and university level and expert knowledge set a solid foundation for international comparison.

**Results:**

The comparative analysis of MoPA in ECTE reveals diverse approaches influenced by national and university policies. A central theme is the variability in MoPA integration across these nations. Finland and Norway prioritize MoPA with independent mandatory courses. In Iceland, compulsory MoPA courses exist at one of two universities, and in Sweden at three out of 19. All university colleges in Denmark offer an elective course. Furthermore, learning objectives related to MoPA are, to varying degrees, part of the internships in the countries, with Sweden being an exception. In the participating countries, the teachers decide the content of the MoPA courses with little guidance, support, and agreement on essential MoPA content within and across the ECTE's. Norway has established guidelines, and in Finland, there is a network of ECTE Physical Education (PE) educators, which, to some degree, increases the consistency and quality of MoPA in education.

**Discussion:**

The Nordic countries present diverse MoPA integration approaches rooted in national policies and educational traditions. The findings emphasize the necessity of independent and mandatory MoPA courses, integration of MoPA into internships and promoting networks across the educational and academic sectors to equip future early childhood educators with competencies for fostering physical activity, motor development and children's well-being.

## 1 Introduction

Movement and physical activity (MoPA) are important components of children's holistic development in early childhood (1). It promotes children's physical and mental health, enhances their cognitive development, and supports their social and emotional well-being (2–6). According to a recent review and meta-analysis, 60% of preschool-aged children (3–5 years) meet the World Health Organization's guideline of engaging in a minimum of 180 minutes of physical activity per day, which includes at least 60 minutes of moderate to vigorous physical activity (7). With the generally high number of young children attending daycare, nursery, kindergarten, preschool, or similar, one way to effectively promote MoPA during the early childhood years is to ensure that teachers and caregivers are adequately trained and supported in this area (8, 9). A recent review conducted by Jones et al. (8) highlighted that consistent and important characteristics of future interventions included providing extra time for children's physical activity each week and involving educators in professional development. Moreover, subgroup analyses in the systematic review by Wick et al. (10) suggest that interventions led by experts yield stronger effects compared to those led by regular childcare teachers, underscoring the essential role of integrating expert knowledge and pedagogic skills in promoting motor skills and physical activity in young children. This is where early childhood teacher education (ECTE) policies come into play.

In the five Nordic countries, Denmark, Finland, Iceland, Norway, and Sweden, almost all children attend early childhood education and care (ECEC) as part of a universal service with well-educated staff (11). As in other countries, ECEC Teachers in the Nordic countries receive less salary compared to Compulsory School Teachers, but are no longer a low-paid group within the public sector (12). There are increased demands imposed by national government policies and parental expectations, which necessitate qualified staff, adequate time and favorable working conditions, which the countries encounter difficulties in fulfilling (12).

In the last decades, there has been a development towards more “education” and a more robust political prioritization of formal learning and evaluation in ECEC (8, 13, 14). Previous research analyzing the values of MoPA using government policy documents (e.g., laws and curricula) on ECEC shows that MoPA in the ECEC policy documents is to varying degrees of a low-priority value in the Nordic ECEC law documents and guidelines (15). Recently, a Swedish study found limited teaching competencies for MoPA in ECEC teachers and highlighted the need for improved education and competence development (16), which has also been found in other countries (17). To study this further, it is important to investigate to what extent MoPA is included and emphasized in ECTE policy documents at the national and university level, setting the direction for future teachers' knowledge, skills, and competencies for teaching and practicing MoPA among young children in the Nordic countries.

This paper aims to provide a comprehensive overview and description of the ECTE policies and regulations related to MoPA in the five Nordic countries. Examining the policies and regulations at the national and university level in these countries increases the understanding of different approaches and identifies areas where improvements can be made. This knowledge can be used to develop ECTE policies and practices that promote MoPA in ECEC within the Nordic countries and globally, considering contextual differences.

## 2 Materials and methods

A shared interest in promoting MoPA in ECEC and at ECTE has led to the establishment of a Nordic research network. The network comprises researchers at eight universities and research institutions from five Nordic countries: Denmark, Finland, Iceland, Norway, and Sweden. The network has previously analyzed policies related to MoPA in ECEC settings (15), and aims to enhance the quality of MoPA in ECTE and ECEC across the Nordic region. In this study, network members have collaborated in investigating policy documents at the national and university levels, which govern the education of ECEC teachers. The study was inspired by the Non-affirmative Theory of Education, which offers a comprehensive approach to understanding multi-level leadership in higher education and how various levels and actors interact. In this view, curricular development in higher education is created in interaction between science, economy, politics, and culture and ranges from the supranational level down to the individual teacher and student level (18). The comparative analytical process can be described in four steps: (1) Delimitation and definitions, (2) Retrieval of documents, initial comparison, and analytical plan, (3) Keyword search, and (4) Qualitative description. During the process, there was an interaction between individual and collective reflection, which unfolded through analytical tasks and discussions within the research network. The process is elaborated in the following.

### 2.1 Delimitation and definitions

The analyses were limited to ECTE specializing in work within ECEC, the education before compulsory education, e.g., a daycare, nursery, kindergarten, or preschool, or other ECEC settings were professionals care for young children. The focus was set on the profession with the highest level of education and thus having the educational and pedagogical responsibility, even though the staff working in ECEC often comprises different disciplines. Two countries provide a master-level education in ECTE (Finland and Iceland). For this comparison, the focus was on the minimum education requirement for working as a teacher with educational and pedagogical responsibility in ECEC. For Finland, this is the bachelor level. For Iceland, this also includes the Master level. The percentages of ECEC staff with the minimum level of education ranges from 40% to 60% for the Nordic countries (12).

We use the term ECTS credits or just “credits”, which indicate the required workload to complete a study program. Each year of full-time study is worth 60 ECTS credits.

MoPA encompasses body movement, physical activity and sensory activities aimed at various purposes, such as fostering the joy of movement, developing skills and confidence in movement, and contributing to cognitive, social, and emotional development (1, 3, 19, 20). In this regard, MoPA aligns with the goals of physical education (PE), which emphasize both learning through movement and using movement as a means of learning (21). MoPA activities in ECEC settings can be structured, guided by ECEC teachers, or unstructured, allowing children to freely explore their environment (22).

### 2.2 Retrieval of documents, initial comparison, and analytical plan

This study retrieved policy documents at the national and university levels that formally regulate the ECTE full-time campus-based programs starting in autumn 2022 (Table 1). Documents used are thus valid for the whole program period, for example, in Sweden, for students from autumn 2022 to the end of autumn 2025. The members of the network were responsible for the retrieval of relevant national and university-level documents. Relevant documents at the national level are ratified documents governing the ECTE, such as laws, ordinances, and official guidelines (Table 1). Documents at the university level are ratified by the university administrations. These documents (curriculums and educational plans) describe the overall ECTE at each university, including a purpose, structure, and requirements. They also entail a directory of courses, a description of internships and the learning objectives of the ECTE program. Internships are structured real-world work experience and exposure to professional environments within ECEC. Internships provide the students with hands-on learning opportunities to gain practical skills and knowledge related to their area of study. Internships can vary in duration ranging from a few weeks to several months, number of credits received, and may be either paid or unpaid (12).

**Table 1:** The structure of early childhood teacher education (ECTE) in Denmark, Finland, Iceland, Norway and Sweden.

Institution type	Denmark	Finland	Iceland	Norway	Sweden
	University college	University	University	University/University college	University
Universities, the total number	6	7	2	12^a^	19
Minimum ECTE requirement	Bachelor	Bachelor	Master	Bachelor	Bachelor
Minimum ECTE requirement, years	3.5	3	5.0	3	3.5
Total workload, ECTS credits	210	180	300	180	210
Compulsory courses, ECTS credits	100	130–134	126–192	115	150–165
Elective courses, ECTS credits	20	25	60/86	50	0-15^b^
Internship, ECTS credits	75	15	38^c^	0 (100 days)	30
Final thesis ECTS credits	15	6–10	10/12 + 0/30^d^	15	15

^a^ Norway has a Rudolf Steiner University College, which not follow the Norwegian law for kindergarten teacher education and is not included in the analyzes.

^b^ Elective courses at 15 credits are given at one university.

^c^ Only the University of Iceland presents internship in terms of ECTS credits.

^d^ BA-theses in Iceland are 10 or 12 ECTS credits. The MT degree does not include a thesis, whereas the MEd degree includes a 30-credit thesis.

Based on an initial comparison of retrieved documents at the national and university level, the network found considerable differences across the Nordic countries regarding the extent of description of the ECTE, which will be elaborated in the results section. In the search for MoPA in policy documents, it became clear that different approaches were used to guide the content and learning objectives of the ECTE. This discrepancy limited the possibilities for direct cross-country comparison. Furthermore, the initial comparison revealed that most documents at the national level did not contain any information related to MoPA. Thus, the analytical plan combined keyword searches and analyses in course names and qualitative content descriptions of the ECTE.

### 2.3 Keyword search and analysis

We conducted a keyword search in the title of all courses at the local university level previously identified as important MoPA words Bod* (body), Coordin* (coordination), Idrott*/liikunta*/Idræt*, Motor*, Move* (movement), Physic* activ* (physical activity) and Physic* educ* (physical education) (15). As the course-level documents were in five different languages, the keywords were identified in each language and then translated into English to facilitate clear, continuous discussions within the research network. The course title keywords underwent independent searches by two researchers for each country. Afterwards, their findings were compared, and any disagreement was confirmed through a second count. Within each country, the number of courses containing MoPA words across the local universities was determined and expressed as the median, maximum, and minimum number of elective and compulsory courses.

### 2.4 Qualitative description

Based on a thorough examination of the national and university policy documents, the syllabuses of the identified ECTE MoPA courses and national expert knowledge of ECTE, a description of the ECTE with a focus on MoPA was provided for each country and compared. This comprised a description of the identified courses and information regarding the integration of MoPA in other parts of the ECTE (other courses and internships). Due to the national differences in structure and policy documents, the information is collected in different ways and dependent on the expert knowledge of the authors. How the study leaders and teachers interpret the policy documents and plan the individual MoPA courses was outside the focus of this study.

## 3 Results

### 3.1 Regulation of early childhood teacher education (ECTE) at the national level

The ECTEs are guided and regulated by different policy documents at national levels in the five countries (Supplementary Table 1). The documents are significantly different across the Nordic countries regarding the extent of description of the ECTE program. In Finland and Iceland, the ECTE's practical implementation is closely connected to the regulation of the Early Childhood Education and Care (ECEC). In Finland, for example, ECTE educators have professional freedoms and opportunities to impact their work (Ministry of Education and Culture, 2016); therefore, they can independently plan, for example, their PE course aims, learning outcomes, and content according to the regulation of the ECEC's [see, e.g. (23),]. In Iceland, a national curriculum document describes five core educational areas in the ECECs but does not describe the specific structure and content of the ECTE. In Denmark and Norway, the Executive Order, and the National Guidelines, respectively, and the learning outcomes of the ECTE are similar. However, universities are free to tone their program for specific profiles within the current law and the ECTE educators are free to choose contents of their courses related to the learning outcomes. In Sweden, the Higher Education Act is the law that regulates universities. ECTE is not explicitly mentioned in the law. The Higher Education Ordinance regulates all higher education in Sweden, such as the ECTE program, and it supplements or clarifies the Higher Education Act. Both the act and the ordinance contain qualitative targets for education. The universities in Sweden have freedom to plan their ECTE program within the current national regulations and thus the structure and courses differs between all universities.

### 3.2 Structure of early childhood teacher education at the university level

Across the Nordic countries, there are different requirements to become an ECEC teacher. The programs in Finland and Norway are at the bachelor level and take 3 years; Sweden and Denmark have 3.5 years of bachelor programs, while a bachelor and a master level education for 5 years is required in Iceland. All countries have internships of different durations as a part of the education.

The different requirements to become an ECEC teacher across the universities in the Nordic countries are elaborated in the following and in Table 1.

- In Denmark, six university colleges offer education programs for pedagogues, with several of them having multiple campuses. After completing the compulsory basic education (70 credits), students choose to specialize in one of three areas—daycare and preschool, leisure and school, or social care and disability. All specializations qualify for working in ECEC. One campus has a specific MoPA profile and integrates movement into all courses without changing the learning objectives of ECTE education.
- In Finland, the education to become an ECEC teacher is possible at seven universities. A bachelor's education qualifies to work as an ECEC and pre-primary education teacher, and a master's education is a requirement for ECEC managers. The bachelor's education includes compulsory courses, electives, and guided teaching practice. Universities can create their own content for study programs (24), leading to minor variations in the structure of courses across the universities.
- In Iceland, two universities are responsible for ECTE. A master's degree is required for employment as an early childhood teacher. Icelandic students first complete a 3-year bachelor's degree followed by a 2-year master's degree including an elective field. The master's degree may include a final thesis (MEd degree) or be without a thesis (MT degree).
- In Norway, 13 universities or university colleges offer ECTE spread across the country on more than 20 campuses. ECTE is based on a national framework plan. In the framework plan, the compulsory areas of knowledge are described with learning outcomes. The individual educational institution must create its own learning outcomes that are based on the national ones, but they cannot be copies of the national ones. Each course is accompanied by an internship, where students carry out practical assignments related to the content of the courses.
- In Sweden, 19 universities are offering the ECTE program. The program is at the bachelor level for 3.5 years. In Sweden, each university needs to have a program syllabus based on the Higher Education Act and the Higher Education Ordinance. The program syllabus regulates the program at the university and must contain the courses within the program, the entry requirements, and other regulations needed. All universities have created their own program syllabus, leading to variations in the structure and content between the universities.

### 3.3 Movement and physical activity in university course titles

The number of compulsory and elective courses at the university level varies substantially across the countries and between the universities within Finland and Sweden (Table 2). The keyword searches in the course titles showed that Finland and Norway have compulsory MoPA courses at 5 or 20 credits at all public universities. In Iceland and Sweden, required MoPA courses exist to a lesser degree (one of two universities in Iceland and three out of 19 universities in Sweden have at least one such course).

**Table 2:** Courses and MoPA courses (MoPA keywords in the title) at the ECTE across the universities in Denmark, Finland, Iceland, Norway, and Sweden.

		Denmark	Finland	Iceland	Norway	Sweden
		Median (Min—Max)^a^				
Compulsory	Total courses, *n*	9 (9–11)	36 (33–70)	12	7	19 (7–27)
	MoPA courses, *n*	0	1 (1–2)	1 (0–1)	1	0 (0–1)
	MoPA courses, credits	0	5 (4–5)	10 (0–10)	20	0 (0–15)
Elective	Eligible courses, *n*^b^	7	NA^d^	NA^e^	5 (0–14)	0 (0–5)
	Selected courses, *n*^c^	1	NA^d^	NA^e^	1 (1–2)	0 (0–1)
	MoPA courses, *n*	1	0 (0–1)	0	0 (0–2)	0
	MoPA courses, credits	20 (10–20)	0 (0–9)	0	30 (20–30)	0

Results are presented as median across universities, and minimum and maximum if there are national differences.

^a^ If variations between universities.

^b^ Number of courses to choose from.

^c^ Number of courses students select.

^d^ In Finland, students have multiple options for elective courses across the university or abroad.

^e^ Number of eligible and selected courses in Iceland vary by university and by educational degree (BEd vs. MEd/MT degrees).

In Denmark, no university college offers compulsory MoPA courses according to the search on MoPA keywords in course titles if the MoPA profile campus is disregarded. All university colleges in Denmark have one MoPA elective courses, while some Norwegian and few universities in Finland offer elective courses containing any keywords. One university in Sweden has elective courses, however not in MoPA, and Iceland has variable number of elective courses but none in MoPA.

### 3.4 Elaborate description of MoPA in ECTE at university level

- In Denmark, students at all university colleges can enroll in the elective course “*Health Promotion and Movement*” corresponding to 10–20 credits. However, this course is across the three specializations in childcare-, school- and afterschool- or the social or special aid area. The course is not offered at all campuses, and the individual students are not guaranteed participation. Around 25%–50% of students are estimated to enroll in the course. The learning objectives in this elective course emphasize strengthening the students’ own movement skills and considering the children's prerequisite for participation in the planned pedagogical activities. During internships, the students should develop competencies to plan, implement and evaluate different educational activities and initiatives for care, health, and prevention. Furthermore, they should be able to motivate and support children's play and aesthetic, artistic, and physical expression. MoPA is, according to the learning outcomes (skills and competencies), also integrated to a small degree in other general courses.
- In Finland, the MoPA course, commonly called “Liikuntakasvatus” (Physical Education), is mandatory in all seven universities offering ECTE programs. Of those seven universities, three also integrate MoPA into other compulsory academic programs, such as music, mathematics, science, and arts. Learning objectives in the MoPA courses in Finland relate, for example, to teaching skills, supporting a child's physical development and health, and enabling learning environments [see also (23)]. The Finnish students are given several options for completing the elective studies (25 ECTS) included in the ECTE degree; however, offered courses vary between the universities. For instance, the 25 elective credits can be completed in individual university-level courses, such as language and communication studies, studies completed abroad or working life studies. Furthermore, minor studies can be completed as one basic study unit, possibly in MoPA-related fields.
- In Iceland, at one of the two universities, there is one mandatory 10-credit course at the bachelor level titled “*Movement and artistic expression, indoors and outdoors*”. No other course titles mention MoPA keywords. The students can specialize in different areas and complete the area chosen with a final thesis, which could be related to MoPA. Furthermore, Iceland has a separate educational track for PE teachers, and the PE education is related to different school levels, although most PE teachers work at post ECEC levels.
- In Norway, all universities and university colleges offer a mandatory 20 credits course of “*Nature, Health and Movement*”. The learning objectives are among others related to children's use of their body and senses in play and learning, motor skills, health-promotion and life skills. There are up till 50 elective credits in the Norwegian model. During the first 2 years of the education the first 20 credits are offered as either one or two different courses. A few institutions offer the opportunity to use all 20 credits aimed at MoPA, while others offer 10 credits in subjects related to MoPA. Some institutions have also chosen to convert 10 or all 20 credits into compulsory subjects. Third year students, at all 12 institutions, have an elective specialization of 30 credits. Most of the institutions offer various forms of physical activity among their specialization subjects. It is important to note that besides the MoPA related keywords all universities offer electives within the area of outdoor pedagogy and outdoor recreation, which have an explicit focus in the Norwegian ECTE.
- In Sweden, there are 338 courses at the 19 universities offering ECTE. Some university courses comprise 30 credits containing sub-courses, which have not been analysed. Two universities have a 7.5 credit course, and one has a 15 credit course with MoPA in the title. These three courses are only partly directed towards MoPA as indicated in the titles such as “*Movement, Nutrition and Health in Preschool*” (7.5 credit*),* “*Early Years Education II, Physical Education and Health*” (7.5 credit) and “*Play, Aesthetics, Creativity and Movement*” (15 credit). There are no elective courses directed at MoPA at the universities in Sweden.

## 4 Discussion

This study explores the integration of MoPA in the policy documents of ECTE in the Nordic countries. A network of researchers from the participating countries conducted a comparative analysis and revealed the varied approaches shaped by national and university policies across the Nordic countries. This difference has an impact on the ECEC teachers' competencies, their prioritizing and motivation for MoPA, and in the end, differences between the MoPA each child meets across ECEC's in the Nordic countries (16, 23). This discussion explores the diverse policies employed in Denmark, Finland, Iceland, Norway, and Sweden while considering overarching themes and their implications. Additionally, it compares the findings with findings from other countries and suggests future directions for development and research within the field of MoPA in ECTE.

### 4.1 Nordic diversity in policies and approaches

Finland and Norway prioritize MoPA at the university level through compulsory courses within their ECTE programs. Although Finland has no binding national policy document obligating it, the universities commonly offer approximately five-credit PE courses. In contrast, Norway has a national framework plan guiding ECTE programs, and each university can make its teaching guidelines based on the framework plan. Finland's “*Physical Education*” and Norway's “*Nature, Health and Movement*” courses exemplify the commitment to standardizing MoPA education. Furthermore, Norway's elective MoPA courses and focus on outdoor pedagogy, enhance competency diversity within the ECEC workforce in Norway. A study of ECTE in Finland revealed that the relevant PE courses predicted pre-service teachers' higher perceived competence in teaching PE (25). However, the university did not influence pre-service teachers' perceived competence in PE, and only minor differences were observed in how PE was delivered between the universities (23). This slight difference in perceived competence could be explained by the compulsory course for PE, similar educational background in MoPA studies for ECTE educators, and collaboration between the educators in Finland. Almost all PE teachers in ECTE programs in Finland have a doctoral-level education in Sports or Health Sciences. Because they form a joint network, it might lead to similar course implementation and high priority of MoPA in ECTE (23).

In Denmark, the elective course option, the lack of specific MoPA compulsory courses, and the limited provision of national guidance and common approaches to MoPA leave a considerable variability in the pre-service teachers' MoPA competencies. In Sweden, there are binding national policy document regulating ECTE, but MoPA is not mentioned in these documents, and at university level, there are even more limited educational opportunities for ECTE students to study MoPA. General education does not offer any or very little MoPA education, which means generally low competence development within the MoPA area (16). In addition, there are no elective courses or specializations in MoPA, meaning no MoPA specialists are trained. Iceland also exhibits limited MoPA offerings, raising questions about the depth of MoPA education.

### 4.2 The role of internships

Internship plays a crucial role in ECTE programs but varies across countries. In Finland, the internship is limited to 15 credit, and findings point to the risk that limited training and supervision may pose challenges for students without prior experience organizing movement and physical activities or creating inspiring learning environments (23). Denmark has the most internship credits, with 75 credits. Iceland and Sweden have 38 and 30 credits, respectively. In Denmark, some learning objectives are directly related to organizing activities and motivating play and physical expression, but it might be ineffective due to the lack of MoPA in other parts of the education. In Norway, internships are closely related to the individual courses. Students plan and deliver smaller practical assignments related to the course's learning objectives, for example, in the course “*Nature, Health and Movement.*” The number and length of internships do not ensure practical competencies within MoPA—especially if MoPA practices are not required during the internship. Supporting students’ perceived competence during their studies becomes essential in these cases to bridge the gap between theory and practice. Culture, role models, responsibility, support, feedback, and transparent integration of MoPA competencies are all essential for the quality of internships.

### 4.3 Essential or optional competencies

The comparative analysis raises the question of whether competencies related to MoPA are crucial or optional for ECEC teachers. In Denmark, Iceland, and Sweden, MoPA is a direct requirement in the law or national curriculum governing the ECEC (15), but it needs to be prioritized to the same degree in the ECTE policies. In Denmark, students *can* choose elective courses and practice the necessary skills during internships, but there are minimal basic requirements. This opportunity seems even smaller in Sweden and in one of the two universities in Iceland. Thus, the students in ECTE do not obtain the necessary competencies to lead and facilitate MoPA in ECEC, which is indisputably essential for the children's development, health, and well-being and as prescribed in the curriculum for ECEC across the Nordic countries (15). The lack of education in MoPA at ECTE may most likely affect the teachers' self-efficacy and motivation to provide high-quality MoPA (16, 23, 26). This can lead to a missed opportunity for children to learn fundamental movement skills and obtain crucial positive experiences in early childhood, resulting in limited engagement in physical activity during later childhood and adulthood with consequences for quality of life and health (2). In these contexts, it is essential to consider ways to ensure that all ECTE students, regardless of program variations, receive adequate knowledge and skills related to MoPA.

### 4.4 The integration of MoPA in ECTE outside the Nordic countries

This paper investigates the integration of MoPA in ECTE in the Nordic countries, and while it appears that the international body of literature has recently increased regarding PE in ECEC it is still scarce on curriculum studies (27). A recent study compared perceptions of PE in ECTE courses in US, Brazil, and Finland. They found that MoPA was a compulsory part of the curriculum in Finland and Brazil, while specific guidelines are lacking in the US (28). This also seems to be the case in Slovenia, where there exists considerable variation in the understanding of PE in ECEC (29), and in Canada, where approximately one third of the newly graduated ECEC teachers have completed MoPA courses (30). Finally, a very recent study from Ankara, Turkey, found that the students improved their initial negative attitudes towards PE during a 16-week required course in *PE and games.* However, it was not sufficient for the students to feel ready to teach PE in ECEC (31). The knowledge of MoPA courses in ECTE internationally is limited, and specifically there is a severe lack of studies evaluating how pre-service teacher education in MoPA leads to higher quality practices in ECEC (27).

### 4.5 Development of MoPA for ECTE

In the Nordic countries, there is a tradition for a high degree of teaching autonomy in higher education, and recent developments towards defined learning objectives and systematic evaluations in ECEC have led to an increased focus on scholastic competencies and less emphasis on care, play, and child-centered holistic child development (8, 13, 14). Considering the scientific evidence underscoring the importance of MoPA for both health and holistic development (1), it is evident that gaps persist within ECTE programs in the Nordic countries. These gaps not only impact the Nordic countries but also hold global significance. On a global scale, UNESCO, stresses the vital role of well-trained early childhood educators in fostering comprehensive child development, including physical well-being (9).

In light of these considerations, future cross-country collaborations are envisioned to establish shared guidelines and frameworks that fortify MoPA in national policy documents and approaches within the Nordic countries. According to the Non-affirmative Theory of Education, understanding higher education leadership requires seeing it as a part of a more extensive dynamic process of creating direction collaboratively, spanning several leadership levels—from international to local levels, and includes a multitude of actors (18). ECTE policymaking is closely connected to ECEC policy and its role and function in society. In this regard, the autonomy of ECTE is limited compared to other higher education, where universities can have greater influence on the curriculum. That said, the ECTE managers and educators in the Nordic countries still experience a large degree of autonomy when it comes to course planning and teaching, which places parts of the leadership at the lower levels. MoPA in ECTE should therefore be considered in a larger context, including dynamic influence from society, culture, academia and actors at different levels of the higher education system, including the ECTE educators (18).

Based on the current study and in line with the theoretical offset, we suggest that ECTE in the Nordic countries should be inspired by best practices to further promote MoPA competencies in ECTE. This includes three areas:

• *Compulsory MoPA*: Establishing a mandatory independent course for PE would ensure that all students get basic skills within the area of MoPA, as seen in Finland (23). Furthermore, it will strengthen the ECTE educators' competencies and lead to professional development, resulting in higher-quality education. Guidelines like in Norway could be established to improve consistency and collaboration across the universities. The guideline gives the ECTE educators a shared understanding when prioritizing content and learning objectives.
• *Integrate MoPA in internships*: Second, internships can play a crucial role in developing MoPA competencies if they are prioritized and connected with other learning objectives of the education. In Denmark and Norway, parts of the internships are linked to MoPA learning outcomes, and the students should obtain experience with planning, implementing, and evaluating different activities.
• *Professional network*: Third, network and collaboration across universities and university colleges within and across the countries should be promoted. In Finland, for example, PE teachers in higher education have formed a network to share knowledge, good practices, and unify teaching content, aiming to enhance the competence of ECEC teachers. These networks could furthermore encompass external stakeholders and the research community to elevate MoPA in ECTE and ECEC.

### 4.6 Strengths and limitations

The comparative analysis across five Nordic countries included close collaboration by national experts and researchers. A combination of investigations of policy documents at the national and university level and expert knowledge set a solid foundation for comparing MoPA in ECTE in those countries. However, there was a notable variation in the analyzed documents across countries, which posed a challenge for direct comparability and hindered systematic comparisons. Consequently, only course titles were employed for the keyword search. The rationale behind this choice was the consideration that if MoPA keywords were not explicitly mentioned in the title of the courses, they might be perceived as having lower educational value in terms of MoPA. It is acknowledged that certain universities may incorporate MoPA elements in specific courses, even if not evident in the course title analyses conducted in this study. In the keyword search, a select set of terms defining the field related to MoPA was utilized. Notably, terms such as “playing,” “nature,” or “outdoor” were deliberately excluded to maintain a focused delineation of the subject matter, recognizing that MoPA might not necessarily be explicitly defined as a target in these courses.

This study focused on the governance of ECTE and was related to the national and institutional levels of the education. We have only to a small degree included perspectives from other levels of the Higher Education Leadership hierarchy (18), and future studies should incorporate perspectives from the ECTE educators and include society and academia in the understanding of the context. This should also include a closer investigation of differences and similarities of the specific MoPA curriculum content and teaching methods.

### 4.7 Conclusion

In investigating the integration of MoPA within ECTE program policies in the five Nordic countries, we found considerable variations. Finland and Norway prioritize MoPA with independent mandatory courses accompanied by elective opportunities. In Iceland, a compulsory MoPA course exists at one of two universities, and in Sweden in three out of 19 universities. The university colleges in Denmark only offer elective courses. Furthermore, practical learning objectives related to MoPA are part of the internships in the countries to varying degrees, with Sweden being an exception. Generally, there needs to be a higher priority, more guidance, and better agreement on essential MoPA content within and across the countries.

This comparative analysis of MoPA in ECTE in the Nordic countries reveals diverse approaches influenced by national and university policies. Given that MoPA is not prioritized in some of the Nordic countries at the national levels of policy and governance, this absence extends to the governing documents and course titles at the university level. This lack of emphasis may lead to an inadequate integration of MoPA in ECTE. To promote quality MoPA education in ECTE, national parliaments must protect children's interests by ensuring that MoPA is incorporated into ECTE policies.

In conclusion, the Nordic countries offer diverse approaches to MoPA integration in ECTE programs, reflecting national policies and educational traditions. The variations underscore the importance of high-level national priority and cross-cultural collaboration to promote quality MoPA education in ECTE and equip future early childhood educators with the competencies to foster physical activity and healthy development among young children.

## Data Availability

The raw data supporting the conclusions of this article will be made available by the authors, without undue reservation.
