# Supplementary material for: A comparative analysis of movement and physical activity in early childhood teacher education policy in five Nordic countries
Source: Front Sports Act Living. 2024 Apr 5;6:1352520. doi: 10.3389/fspor.2024.1352520 (PMC11026603; doi:10.3389/fspor.2024.1352520)
Supplement: Supplementary file 1 [file Table1.pdf]

Supplementary Table 1

ECTE (and ECEC) policy documents at the national level in Denmark, Finland, Iceland, Norway and Sweden. All documents assessed Autumn 2022.

	Denmark	Finland	Iceland		Norway		Sweden	
**Document type**	**Law**	**Law**	**Law**	**National Guidelines**	**Law**	**National Guidelines**	**Law**	**Ordinance**
Name of document, Original language	Bekendtgørelse om uddannelsen til professionsbachelor som pædagog	Yliopistolaki 558/2009<br>Varhaiskasvatuslaki (540/218)	Reglugerð um inntak menntunar leik-, grunn- og framhaldsskólakennara	Aðalnámsskrá leikskóla	Forskrift om endring i forskrift om rammeplan for barnehage-lærerutdanning	Forskrift om rammeplan for barnehage-lærerutdanning	Högskolelag (1992:1434)	Högskoleförordning (1993:100)
Name of document, English	Executive Order on the education for professional bachelor as a pedagogue	Universities Act 558/2009<br>Act on Early Childhood Education and Care (540/2018)	Regulation on the content of the education of preschool, primary school and secondary school teachers.	The Icelandic National Curriculum Guide for Preschools	Regulations on changes to the regulations on the framework plan for kindergarten teacher training	National guidelines for early childhood teacher education	The Swedish Higher Education Act (1992:1434)	The Higher Education Ordinance (1993:100)
Link to document	https://www.retsinformation.dk/eli/lta/2017/354	https://www.finlex.fi/en/laki/kaannokset/2009/en20090558<br>https://www.finlex.fi/fi/laki/ajantasa/2018/20180540#L6P26	https://www.stjornartidindi.is/Advert.aspx?ID=664c0147-f0b5-44db-8c7a-23b4f14e3b6e	https://www.government.is/library/01-Ministries/Ministry-of-Education/Curriculum/adskr_leiksk_ens_2012.pdf	https://lovdata.no/dokument/LTI/forskrift/2022-12-21-2542	https://www.uhr.no/_f/p1/ia6b16fb1-45bf-432e-afdd-79e9c3c40e76/41738_1_a.pdf	https://www.riksdagen.se/sv/dokument-lagar/dokument/svensk-forfattningssamling/hogskolelag-19921434_sfs-1992-1434	https://www.riksdagen.se/sv/dokument-lagar/okument/svensk-forfattningssamling/hogskoleforordning-1993100_sfs-1993-100
Latest version of document	2017	2017<br>2018	2009	2011	2022	2018	2021	2022
